# Opportunities and Challenges for the Emerging Field of Positive Emotion Regulation: A Commentary on the Special Edition on Positive Emotions and Cognitions in Clinical Psychology

**DOI:** 10.1007/s10608-017-9831-3

**Published:** 2017-02-23

**Authors:** Barnaby D. Dunn

**Affiliations:** Mood Disorders Centre, University of Exeter, Exeter, EX4 4QG UK

## Abstract

The importance of developing a better understanding of positive emotion regulation in both healthy and clinical populations is now recognised. This special edition brings together leading figures in the positive emotion regulation field and has contributions characterizing positive phenomena, differentiating them from negative phenomena, and evaluating underlying psychological mechanisms that drive these phenomena. This commentary reviews these articles to highlight challenges and opportunities for this emerging field, including the need to better characterize positive phenomena, to be more explicit about how the links between negative and positive phenomena are conceptualised, to evaluate more robustly underlying mechanisms, to standardize measurement of positive constructs, and to ensure that these scientific findings lead to meaningful changes in real-world policy and practice.

Emotion regulation is the variety of processes used to change the nature, frequency and intensity of emotion experience (Gross 2015). Historically, basic science and clinical research have focused on understanding the consequences of emotion regulation strategies that aim to modulate negative feelings and how these may impact on psychopathology. However, more recently, there has been an increasing recognition that the regulation of positive feelings is also important (Bryant et al. 2011; Carl et al. 2013; Quoidbach et al. 2015; Hofmann et al. 2012). Understanding how to modulate positive emotions in a context appropriate fashion could not only increase levels of wellbeing in the general population but also lead to better outcomes for clinical disorders characterized by abnormalities in positive affect regulation. These include those conditions characterized by reduced positive affect (anhedonia), such as depression, social phobia and schizophrenia (e.g.; Dunn 2012; Dunn and Roberts 2016; Kashdan et al. 2011; Watson and Naragon-Gainey 2010) and those conditions characterized by elevated positive affect, such as the manic phase of bipolar disorder (e.g., Gruber 2011).

This special edition brings together leading basic science and clinical researchers in this emerging field in an effort to improve understanding and treatment of positive emotion regulation in psychopathology. This commentary on the special edition will use the articles in the special edition to highlight challenges and opportunities for the field of positive emotion regulation and to suggest some future directions of travel. It will in turn focus on: how best to operationalize positive constructs; whether positive and negative phenomena are best characterized as orthogonal to one other; how to characterise underlying psychological mechanisms driving positive change; how to measure positive change; and finally how to ensure key findings are translated into meaningful changes to policy and practice.

## Characterizing Positive Phenomena

Many of the articles in this special edition represent good illustrations of how to pay the same degree of attention to positive phenomena that has historically been paid to negative phenomena. The study by Gruber and colleagues illustrates the benefits of a fine grained characterisation of positive (and negative) emotions in terms of differentiating between risk for mania and depression (Gruber et al. 2016). In a sample of outpatient adolescents, higher self reported joy and contempt were uniquely associated with mania symptoms (over and above depression symptoms). In contrast, low levels of joy and high levels of sadness were uniquely linked to depression (over and above mania symptoms). This is interesting on a number of levels. First, it demonstrates the importance of balance in affective experience. In particular, the same emotion (joy) was linked to mania at high levels and depression at low levels, presumably indicating that a moderate level of joy is associated with optimal wellbeing. Second, it illustrates that blends of positive and negative emotion can most helpfully distinguish between symptoms of different forms of psychopathology. What would now be interesting to explore is whether similar patterns of affective disturbance are linked to psychopathology across the life span (adolescence, adulthood and older age) and whether these patterns change with chronicity (first onset versus repeat episodes). For example, it is conceivable that repeated experience of the detrimental consequences of a manic state could mean that in mania individuals increasingly come to fear positive emotions (e.g. Edge et al. 2013) so elevated joy may be a less significant marker of bipolar disorder with chronicity.

It is also important to characterize positive phenomena beyond mere affect, including positive cognition and positive behaviour. The study by Rice and Fredrickson (2016) in this special edition demonstrates how spontaneous positive thoughts are more likely to occur for things towards which we feel harmonious passion (those that we internalize autonomously and that are not in conflict with other roles we hold; Vallerand et al. 2003). Further, these spontaneous positive thoughts make it more likely that behaviour consistent with this passion will be adopted. This study has parallels with an existing literature on spontaneous memories and prospection that has long recognised that individuals tend to have spontaneous remembrances of positive events in the past and spontaneous imaginings of a positive future (e.g., Berntsen and Jacobsen 2008). These experiences are more likely to emerge in situations that are low in attentional demands, are often prompted or triggered by an environmental cue, tend to have a high degree of distinctiveness and specificity, and lead to a distinct impact on mood (see Berntsen 2007 for a review). Interestingly, imaginings of the future tend to be more positive and idealistic than remembrances of the past (Bernsten and Jacobsen 2008). Moreover, in the clinical domain the characteristics of positive and negative intrusive memories have begun to be contrasted (Moulds et al. 2012). It will be important for future work to delineate the extent to which positive spontaneous thought is a distinct process, rather than one which overlaps with positive mental time travel to the personal past or future. There is potential for developing novel intervention techniques that capitalise on these insights, for example encouraging clients who struggle to experience positive phenomena to place themselves in situations that maximise the likelihood of spontaneous positive thought occurring.

There is also likely to be an interaction between these positive feelings, cognitions and behaviours and the environment, which could be capitalised on therapeutically. Interesting in this regard is the study by Disabato and colleagues in the special edition, which demonstrates that possessing particular personality strengths (gratitude and meaning in life) makes individuals more likely to experience subsequent positive life events (Disabato et al. 2016). The authors argue that these personality strengths motivated individuals to engage in behaviours that make the positive life events more likely to occur (for example, promoting prosocial behaviours that lead to subsequent rewards). Surprisingly, these increases in positive life events only mediated the association between these personality strengths and improvement in depression symptoms over three (but not six) months. The authors explain this in terms of hedonic adaptation, such that over time the same activities no longer generate the same degree of positive affect (Diener et al. 2006). However, this account would only logically hold if the individuals were completing identical or very similar positive activities across the six month period. It seems likely that individuals to some extent vary their repertoire of positive activities over time. Either way, it is interesting to have linked the concept of the hedonic treadmill to the outcomes of positive activity scheduling. This has implications for the delivery of treatments like Behavioural Activation (where the central intervention techniques are overcoming avoidance and scheduling positive activities) to bolster positive affect. While behavioural activation approaches have been shown to bolster wellbeing (Mazzucchelli et al. 2010), there is clearly room for improvement. It may be helpful to encourage individuals explicitly to build in variety to the activities they schedule to minimise hedonic adaptation, and thus maximizing the potency of behavioural activation to bolster positive mood over time.

Finally, the study by Coughlan, Tata and MacLeod (2016) demonstrates how a positive outlook may not always be beneficial, in this instance in the prospective domain. They examine why deliberately self-harming individuals tend to remain over-invested in goals they believe are unattainable (a processing style that exacerbates distress in the longer term). One possible explanation is that individuals who self harm focus on the degree of wellbeing they will feel should they achieve the goal, rather than on the steps needed to complete the goal (cf. Oettingen 2012). As expected, the study found that the deliberate self-harm group had significantly lower levels of current well-being than a control group. However, there was no difference between the groups when predicting their future well-being if they attained positive future goals (i.e. imagined goal attainment). This study is part of a broader literature highlighting the potency (for better and for worse) of prospection (Roepke and Seligman 2016). There is an increasing focus in interventions on modifying prospection instead of or as well as memory, for example Future Directed Thinking (Vilhauer 2014).

In summary, the articles in the special edition demonstrate how positive affective, cognitive and behavioural phenomena in both clinical and non-clinical groups may be characterized more precisely. This is the first critical step in correcting disturbances in positivity seen in psychopathology.

## Characterizing the Interrelationship Between Positive and Negative Emotion Regulation

Perhaps the key challenge for the positive emotion regulation field is to reach consensus about how to integrate with the large body of work looking at negative emotion regulation. A number of articles in the special edition directly speak to this issue or are implicitly influenced by it.

Johnson and Wood (2015) review how the positive psychology movement emerged as a counter-point to the pathological focus in classic clinical psychology. They argue that this has led to an unhelpful fractionation of the field, partly driven by market pressures, into seeing ‘positive’ and ‘negative’ phenomena as distinct from one another. They convincingly argue that what are instead needed are conceptual frameworks and intervention products that simultaneously aim to reduce the negative and build the positive—referred to as a ‘positive clinical psychology’ approach (Wood and Tarrier 2010). It is difficult to disagree with this analysis in its moderate form (see also Hofman et al. 2012; Hofmann 2016).

Johnson and Wood go further than this, however, to make the claim that positive and negative constructs should universally be conceptualised as lying at opposite ends of the same continuum. This is a more controversial argument. If true, this has potentially important clinical implications, since it would suggest that the same intervention approaches should be both able to reduce negative and increase positive mood. There are some challenges to the stronger form of this argument, however. For example, the fact that individuals report being able to experience ambivalent emotional experience (simultaneously experiencing positive and negative emotions at the same time and sometimes to the same object; e.g. Corradi 2013; Rees et al. 2013), cannot easily be accounted for by continua arguments. Moreover, there is emerging evidence that manipulating the same underlying psychological mechanism can increase both positive and negative emotion experience. For example, the tendency to be aware of sensory and bodily experience (‘observing’) has been associated with mixed outcomes in the mindfulness literature, such that it is linked to increases in wellbeing and in levels of psychopathology (e.g. Baer et al. 2008; Williams et al. 2014). One explanation is that this tendency to observe serves as a global (positive and negative) affective amplifier. Such an explanation is hard to reconcile with a view that positive and negative emotions are on opposite ends of the same continuum (as increasing one should decrease the other). Of course, there are other possible accounts (for example, that the ‘observing’ mechanism plays out differently as a function of meditation experience; Williams et al. 2014).

The continuum perspective can be contrasted to views that positive and negative are at least partly orthogonal constructs. In affective neuroscience, the case has been made that the systems that drive approach to rewarding stimuli (associated with positive affect) are to some extent dissociable from those that drive withdrawal from punishing stimuli (associated with negative affect) (Watson et al. 1988; Gray 1987; BIS/BAS). In the sociology literature, the case has been made that illbeing is largely independent from wellbeing, such that it is possible to experience pockets of wellbeing even in the midst of illbeing (Keyes 2005). Of course, a hybrid position is possible, so that some positive and negative phenomena lie on opposite ends of single continuum (and are driven by common underlying psychological mechanisms), whereas other positive and negative phenomena may be genuinely distinct. In many ways this is an optimistic message for healthcare, for example suggesting that clients suffering from chronic physical health conditions (high in illbeing) may still experience intervals of wellbeing. Moreover, it opens the door to a personalized medicine approach, such that individuals high in illbeing may benefit from one kind of treatment approach, whereas those characterised by low wellbeing may benefit from an entirely different treatment. Emerging analytical techniques have the potential to match the right patient to the right treatment in this way, for example the work on the personalized advantage index by DeRubeis and colleagues (DeRubeis et al. 2013).

The way in which the interrelationships between positive and negative phenomena are conceptualised can substantively alter interpretation of studies. For example, the interesting study by Trompetter et al. (2016) in this special edition provides cross-sectional support for the importance of developing self-compassion in both reducing psychopathology and increased positive mental health. This is part of an exciting emerging literature on the beneficial effects of cultivating self-compassion (for reviews, see Strauss et al. 2016; Leaviss and Uttley 2015). How the Trompetter et al. data are interpreted critically, however, depends on the implicit underpinning conceptualisation of how positive and negative phenomena interrelate. The authors arguably are viewing mental health (‘positive’ phenomena) and psychopathology (‘negative’ phenomena) as conceptually separable constructs, given that they argue mental health drives reduced psychopathology. However, if increased positive mental health and decreased psychopathology are simply two ends of the same underlying continuum (and so are measuring the same underlying construct), it is unsurprising that when one end of the continuum is partialled out the other end of the continuum is no longer linked to compassion. It is not possible for cross-sectional data of this kind to differentiate between these alternative explanations, so there is a need for further work using different methodological approaches to distinguish between them.

The choice of underlying conceptual framework can also influence the design of interventions, potentially impacting on the eventual efficacy of the treatment. For example, the Chaves et al. (2016) study in this special edition report results of an interesting controlled trial contrasting the benefits of group cognitive behavioural therapy to a bespoke positive psychology group programme, finding both treatments were comparably partially effective for treating depression. From a perspective that positive and negative phenomena are to some extent orthogonal it could be argued that it is not surprising that there was no clear difference between these treatments (and that both did not lead to optimal recovery). The CBT group would be expected to target repairing elevations in negative affect. The bespoke positive psychology group would be expected to target repairing reductions in positive affect. To lead to full recovery, both of these would need to be treated by a single intervention. From this vantage point, it would be interesting for this study to be repeated with a third treatment arm that simultaneously targets repairing positive and negative affect, with the hypothesis being that this would be superior to both of the other treatments. However, a note of caution is required here. There was no evidence of superiority of CBT over the positive group at repairing negative affect constructs and nor was there any evidence of superiority of the positive group over CBT at repairing positive affect.

In summary, it is likely to be helpful for the field going forwards if studies are explicit about their underlying theoretical conceptualisation of how positive and negative affect systems are interrelated.

## Characterizing the Psychological Mechanisms Underpinning Positive Phenomena

The focus of many of the articles in this special edition is to characterize what underlying psychological mechanisms drive alterations in positive phenomena. The thrust of the Trompetter et al. (2016) study discussed above is that increases in positive mental health lead to reductions in psychopathology, which is mediated by an increase in self-compassion. However, the authors also acknowledge that the reverse mediation holds, meaning that the cultivation of self-compassion is likely to be a promising approach to build positive mental health.

Jazaieri et al. (2016) examine whether changes in two forms of emotion regulation—expression suppression and reappraisal—account for the beneficial effects of CBT for social phobia on life satisfaction measures in a randomized controlled trial. As expected, the CBT arm showed a greater increase in reappraisal and life satisfaction, relative to a wait list control group. Unexpectedly, CBT did not lead to a greater reduction in expression suppression in the CBT relative to the control condition. The degree of change in reappraisal in the CBT arm was not significantly related to change in life satisfaction. However, greater decrease in expression suppression did correlate with a greater increase in life satisfaction. It is difficult to interpret the lack of a significant correlation between reappraisal change and life satisfaction change. It may be that reappraisal change is genuinely unrelated to life satisfaction change (i.e. is not an active mechanism of the therapy). Alternatively, it could be that there was insufficient variability in reappraisal change to be able to meaningfully predict individual differences in life satisfaction change. This study also demonstrates some of the challenges in trying to establish mechanism of action in a therapy. Change in each emotion regulation mechanism and the life satisfaction outcome variable were calculated over identical time scales, meaning that the temporal precedence criterion necessary to establish mediation is not met (cf. Kraemer et al. 2002). A more powerful design would have been to assess early change in the emotion regulation mechanism and see whether this predicts subsequent change in life satisfaction. However, this presupposes the time scale over which change in therapy occurs (i.e. an early change in mechanism leads to a later change in outcome) and this pattern may vary from individual to individual. There are currently very few studies in the psychological therapies outcome literature that have been fully able to meet the criteria necessary to establish mediation beyond doubt because of these challenges.

The study by Garland and colleagues demonstrates another way to examine the mechanisms of action of an active treatment (Garland et al. 2016). They explore the impact of mindfulness-based practice on eudemonic wellbeing, measuring all constructs weekly during the course of treatment to enable time lagged analyses (whether mechanism at time *n* predicts outcome at time *n* + 1 and vice versa). The study demonstrates that state mindfulness and positive appraisal (making positive sense of challenging events) work together in an upward spiral to build eudemonic wellbeing. As the authors acknowledge, an important next step is to attempt to simultaneously examine the upward spiral between state mindfulness and both hedonic and eudemonic measures of wellbeing, given increasing evidence that mindfulness also increases positive emotion experience (Geschwind et al. 2011). Taking weekly mechanism measures makes it possible to test potential mediating effects of a treatment more robustly, although does increase the testing burden on participants and it will not be possible to measure every possible mediating mechanism in such a fine grained way.

Vanderlind and colleagues examine what underlying psychological mechanisms may prevent depressed individuals being able to repair negative affect via the recall of positive autobiographical memories (Vanderlind et al. 2016). Participants were asked to undergo a negative mood induction and then to try and repair the negative mood generated by recalling a positive personal memory. Those individuals who reported a greater trait tendency to be afraid of positive emotion experience (for example, that feeling good would make them lose control), were less able to increase positive mood and decrease positive mood during the memory recall. Had fear of positive affect been measured using a state rather than trait scale, it is conceivable that this relationship would have been even stronger. While this study did not use a clinical sample and there was a limited spread of depression severity, there was some support for the theory that fear of positive affect mediated the relationship between depression severity and a reduced capacity to repair negative mood via positive memory recall. This study builds on an emerging literature linking fear of positive affect (and related constructs like the utilisation of dampening appraisals to inhibit positive affect) to both anhedonia and depression. For example, fear of positive affect and dampening appraisals have been associated with anhedonia in cross-sectional studies (Werner-Seidler et al. 2013). Moreover, manipulating the use of dampening appraisals has recently been shown to lead to drops in positive affect and increases in negative affect during positive memory recall (Burr et al. 2017). Arguably, fear of positive affect is now a strong candidate mechanism driving anhedonia to target in clinical interventions.

There is now reasonably convincing evidence that the tendency to engage in an experiential mode of processing (characterized by attending to sensory and bodily experience) is associated with increased positive affect. For example, imagining scenarios in the mind’s eye as opposed to thinking about them verbally enhances positive affective experience in laboratory studies (Holmes et al. 2009). Moreover, clinical trials demonstrate that imagery training can help repair anhedonia in the context of depression (see Holmes et al. 2016 for a review). The study in the special edition by Renner and colleagues further extends this literature by showing that imagery makes individuals more likely to engage in subsequent positive activities (Renner et al. 2016). Conducting a secondary analysis of the randomized controlled trial (Blackwell et al. 2015), they found that individuals in the imagery condition showed a faster increase in levels of behavioural activation compared to those in the non-imagery active control condition. This is remarkable given that the imagery training (resolving ambiguous everyday scenarios in a positive direction) did not specifically ask individuals to engage in imagery about positive activities coming up for them in the near future. A logical next step is to see if asking individuals to imagine scheduled activities in the next week enhances their likelihood of engaging with them. It would also be interesting to see whether this increase in behavioural activation in part accounts for why imagery training leads to a reduction in anhedonia (cf. Blackwell et al. 2015).

In summary, articles in this special edition garner further evidence that positive phenomena can be cultivated in a variety of ways, including via cultivating experiential processing, positive appraisal style and self-compassion and reducing fear of positive affect. Further clarifying which mechanisms influence positive phenomena is of key importance to this emerging field. Early positive psychology approaches were arguably mechanisms ‘neutral’, focusing on how particular intervention techniques could boost positive mood and reduce psychopathology but not explicitly relating this back to an underlying theoretical framework as to how these benefits may come about. This limits the potential to identify the critical active ingredients of a positive therapeutic response and also makes it harder to determine which treatments work best for which individuals (the personalized medicine agenda; e.g. see DeRubeis et al. 2013).

While studies such as those included in this special edition represent significant progress in this regard, it is important to acknowledge that this is an embryonic literature and no underlying mechanism has currently been fully supported. To ensure that positive psychology and wellbeing research is taken seriously by academics, clinicians, and policy makers, it is important that the bar for what counts as ‘good enough’ evidence for a mechanism is set high. The following criteria can be used to establish a mechanism as a strong candidate to be targeted in intervention. First, the mechanism should be cross-sectionally associated with the outcome of interest. Second, the mechanism measured at time one should predict change in the outcome from time one to time two in prospective designs. Third, manipulating the mechanisms in both tightly controlled laboratory conditions and more ecologically valid settings should change the outcome in the expected direction. Fourth, change in the mechanism should be related to improvements in the outcome in psychological therapies, ideally this being established using robust mediation designs that meet the criteria recommended by Kraemer and colleagues (Kraemer et al. 2002). Finally, given ongoing concerns about the lack of replicability of findings in psychology generally (see Open Science Collaboration 2015), it is also important to demonstrate an effect more than once, ideally across different laboratories (to minimise any allegiance biases) and triangulating across different measures (to ensure that any effect is not simply an artefact of the specific method adopted). At the present time, as far as I am aware, no mechanism in the positive psychology and wellbeing literature fully meets these criteria and it would be beneficial for the field to aim to make this happen.

## Characterizing how Best to Operationalize Positive Constructs

The range of different ways that positivity is operationalized across the studies in this special edition is noteworthy. For example, to index behavioural activation, the Behavioural Activation for Depression Scale (BADS; Kanter et al. 2007) is used by Renner et al. (2016). To measure positive mental health, Trompetter et al. (2016) apply the Mental Health Continuum Short Form (MHC-SF; Keyes 2002) – a composite index of emotional, psychological and social wellbeing. To index passion for a given activity, Rice and Fredrickson (2016) use the Passion Scale (Vallerand et al. 2003) to measure harmonious and obsessive passion. Jazaieri et al. (2016) measure life satisfaction using the Satisfaction with Life Scale (WSLS; Diener et al. 1985). The focus in the Vanderlind et al. (2016) study is positive hedonic experience, measured using a single item happiness rating scale.

In each case, these choices of measure seem appropriate and well considered. However, this proliferation of measures in the field presents challenges when trying to generalise across studies. The constructs measured are likely to share common ground but also to differ in important ways. This makes it difficult to infer commonalities across studies and limits opportunity for meaningful replication. This problem has been stated most forcibly by Sally Davies (at the time the Chief Medical Officer of the UK): “it seems clear from the outset that ‘wellbeing’ means different things to different people. Each approach has inherent strengths and weaknesses, but one thing is obvious: there is no clear consensus in the best way to define and measure well-being within mental health.....” (p. 36, Annual report of the Chief Medical Officer 2013).

This is a key challenge for the field, particularly in the face of some sceptical audiences who prefer a psychopathology lens and see positive focused approaches as ‘soft science’. It would be useful to agree standard measures for the field to use and include these where appropriate in studies. Any measure that is selected needs to be well validated and ideally to have general population normative data. For example, a measure of reduced hedonic experience in the clinical domain could be the Snaith Hamilton Pleasure Scale (SHAPS; Snaith et al. 1995), which, it has been argued, is as close to a gold standard measure of anhedonia as currently exists (Rizvi et al. 2016). As a broader measure of wellbeing (combining eudemonic and hedonic wellbeing), the Warwick Edinburgh Mental Wellbeing Scale (WEMWEBS; Tennant et al. 2007) shows promise.

In addition to agreeing standard measures of positive functioning and wellbeing, it is also useful for studies in the mental health domain to combine these measures with additional ones indexing psychopathology. This makes it possible to further examine the extent to which these are orthogonal constructs (as argued by Keyes 2005) or whether they are at opposite ends of a continuum of functioning (as argued by Johnson and Wood 2015). In this regards, the study by Chaves and colleagues in the special edition is a good example, as it includes a diverse array of both positive and psychopathological measures (Chaves et al. 2016).

This commentary will finish with some general remarks about how to increase the likelihood that science of this kind leads to direct practical application.

## The Importance of Co-design and an Implementation Science Perspective

It is critical for the positive emotion regulation field not to lose sight of the need for practical application of its findings. It has long been acknowledged that a disappointingly small proportion of basic science findings lead to the development of novel treatments or changes in healthcare practices (Grimshaw et al. 2012). The Cooksey report in the UK identified two key gaps in the translational pipeline: a failure to translate basic science findings into the development of new health care products and the failure of effective health care products to be routinely implemented in standard practice (Cooksey, 2006). This is in part driven by ‘funding gaps’ that exist. For example, there is relatively little funding to support researchers in the early development of an intervention that is necessary before it can be taken to clinical trial. It is also partly driven by a move towards ‘silo-isation’ and over specialisation. For example, individuals with an interest in both academic research and clinical practice are often forced by systemic pressures to focus on one over the other. This is unfortunate, as individuals who are still actively engaged in both research and health care delivery are likely to have a clearer vision about which basic science questions are important for the healthcare community and how to then translate the answers to these questions into practical change.

Even when research is translated, this is often a slow and torturous process – for example, it is estimated it takes around 17 years for something to move from bench to bedside (Morris et al. 2011). There is a danger that at the end of this process, the intervention is no longer fit for practice, as the health care context may have changed. For example, an intervention that may have been seen as affordable in a United Kingdom National Health Service before the global financial crisis, may no longer be affordable after this time. The challenge for positive psychology approaches is both to ensure translation happens and that this proceeds at a sufficient pace so that the end product still fits the context for which it was designed.

One key way to maximize the likelihood that positive psychology research moves across the full translational pipeline to influence health care delivery is routinely to engage in widespread stakeholder consultation. It is crucial that a positive clinical psychology intervention is acceptable to all key parties, potentially including the service-users who receive them, the clinicians who deliver them, and the mental health service commissioners that pay for them (in countries where there is a national mental health service model). It is noteworthy that none of the articles in this special edition explicitly mention these stakeholder perspectives. While many of these interventions and experiments are likely to have been influenced by these stakeholder views, will benefit the field to hold this at the front of the mind and to be explicit when writing findings up. These stakeholder views should ideally be consulted throughout the research pipeline, from basic science through intervention development to intervention implementation. There is a particular danger with positive clinical psychology approaches that they may come across as “Pollyanna-ish” (Dunn 2012) – in other words naively positive and too good to be true. This could potentially lead to service-users disengaging from them and clinicians not wanting to deliver them. There may be cultural differences in how clients react to these interventions and it is important to take these into account when applying interventions in new contexts if there is desire to move to a cross-cultural positive clinical psychology (cf. Johnson and Wood 2015). The intervention mapping framework – an approach pioneered in health psychology to aid the effective development and roll out of public health interventions – emphasises the importance of this co-design process and could be a useful guiding heuristic for the field to use (Bartholomew Eldrigde et al. 2016).

A critical issue in the uptake of positive clinical psychology interventions will also be their affordability. With an ageing population and an increasing array of expensive intervention approaches that can be used to manage chronic diseases, governments have to make hard decisions about how to ration health care. Interesting in this regard is the choice of delivery format in the various interventions reported in this special edition. For example, Chaves et al. (2016) have developed a group positive intervention, which is likely to be more cost effective than individual therapy options. Renner et al. (2016) report a secondary analysis of an internet delivered imagery training. After initial development costs have been accounted for, such approaches are likely to be highly cost-effective. There is little evidence of a reduction in clinical efficacy when moving from individual to group therapy (for example, Wergeland et al. 2014) or from face-to-face to internet delivered protocols (Andersson et al. 2014), so such approaches are increasingly attractive from an economic perspective. However, service-users continue to hold mixed views about the acceptability of e mental-health approaches (Musiat et al. 2014), so there may be high levels of refusal of treatment or drop out. This may change with the increasing familiarity with a digital world, particularly in younger service-users. However, there is good evidence that some degree of support is needed to maximise the effects of these interventions, so a guided self help approach is more likely to be useful than a fully self-guided internet approach (Saddichha et al. 2014).

Implementation science perspectives may also maximise the translation of positive findings. In particular, by thinking from the outset about the likely implementation of a novel positive intervention, this increases the chance that it will be widely adopted by potential users. For example, a helpful framework in this regard is Normalisation Process Theory (May 2013), which discusses ways to ensure that a health care intervention becomes a routine part of clinical practice over the long term. In particular, it is useful to think of the sense-making people do when first tasked with implementing a new practice, the relational work that is necessary to build and sustain a community of practice around a complex intervention, the operational work that helps individuals enact a set of practices, and the appraisal work that individuals do when evaluating the impacts of a set of practices on themselves and others.

## Conclusions

This special edition has a range of interesting contributions that help characterize positive phenomena, explore how interrelated to negative phenomena they are, and identify psychological mechanisms underlying positive phenomena. These articles represent excellent examples of an emerging body of work on positive emotion regulation and its impact on psychopathology. To ensure the ongoing growth of this burgeoning field and to help it make a meaningful practical difference, it will be beneficial to measure positive constructs more robustly, to increase the rigor with which underlying mechanisms driving positive phenomena are evaluated, and to adopt an implementation science perspective to ensure findings are carried into real world practice.
